# Patient-matched transcriptomics of in vivo and in vitro colonic epithelium substantiate organoids as a translational model for ulcerative colitis

**DOI:** 10.1038/s41598-026-54857-7

**Published:** 2026-06-14

**Authors:** Gunnar Andreas Enerhaug Walaas, Arun Sridhar, Lusie Frostvoll Kuraas, Siri Sæterstad, Amanda Yin Schioldborg, Atle Van Beelen Granlund, Arne Kristian Sandvik, Ann Elisabet Østvik, Ingunn Bakke, Torunn Bruland

**Affiliations:** 1 Department of Clinical and Molecular Medicine (IKOM), Faculty of Medicine and Health Sciences, NTNU-Norwegian University of Science and Technology, Prinsesse Kristinas gt. 1, 7030 Trondheim, Norway; 2 Department of Gastroenterology and Hepatology, Clinic of Medicine, St. Olav’s University Hospital, Trondheim, Norway; 3 Clinic of Laboratory Medicine, St. Olav’s University Hospital, Trondheim, Norway; 4 Department of Pathology, St. Olav’s University Hospital, Trondheim, Norway

**Keywords:** Inflammatory bowel disease, Colonoids, Precision medicine, Epithelial inflammation, Disease modeling, Computational biology and bioinformatics, Diseases, Gastroenterology, Immunology

## Abstract

Ulcerative colitis (UC) is characterized by cytokine-driven inflammation and barrier disruption in the colon, making epithelial dysfunction central to disease pathology. Intestinal epithelial organoids (IEOs) preserve donor-specific genetics and architecture, offering a promising model, but their ability to replicate patient-specific epithelial inflammation remains undetermined. To directly compare in vivo epithelial transcriptional states with defined cytokine-induced responses in vitro, we analyzed patient-matched transcriptomics from laser microdissected inflamed and uninflamed colonic epithelium and IEOs derived from uninflamed biopsies of the same UC patients. IEOs were stimulated with UC-relevant cytokines (TNF, IFNγ, IFNλ1, or TNF + IFNγ) or a cytokine cocktail (TNF, IL17, IL1β, IL22, Poly(I:C), IFNγ). Key inflammatory genes were validated by immunoblotting and immunostaining. Cytokine-stimulated IEOs recapitulated key in vivo epithelial inflammation, including interferon signaling, antigen presentation, and unfolded protein response pathways. Among the tested conditions, TNF + IFNγ combination and the cytokine cocktail most closely replicated UC epithelial inflammation, with concordance for over 350 UC-relevant genes and protein-level validation of IRF1, ERAP2, NOS2, DUOX2 confirmed patient-dependent expression between inflamed epithelium and cytokine-stimulated IEOs. Our study shows that cytokine-stimulated IEOs provide a robust, personalized platform for modeling epithelial inflammation, enabling discovery of epithelial-specific disease mechanisms and therapeutic targets.

**Supplementary Information:**

The online version contains supplementary material available at https://doi.org/10.1038/s41598-026-54857-7.

## Introduction

Ulcerative colitis (UC) is a chronic inflammatory bowel disease (IBD) affecting millions worldwide with increasing incidence and prevalence^1^. It typically manifests in young adults and, without a cure, causes decades of disease burden^2^. UC has a multifactorial etiology, with genetic predisposition, environmental triggers, microbial dysbiosis, and immune dysregulation contributing to colonic mucosal inflammation. The UC immune response comprises neutrophil infiltration^3^, a type 17 T helper (Th) cell response with increased secretion of interferon-γ (IFNγ), tumor necrosis factor (TNF), and interleukin (IL)17^4,5^, and humoral responses^6^. A key characteristic of UC pathophysiology is a shift in the balance of pro- and anti-inflammatory cytokines and breakdown of the epithelial barrier causing pathogenic interactions between lamina propria resident immune cells and the microbiome.

The fundamental role of cytokines in UC inflammation is supported by identification of single-nucleotide polymorphisms in cytokine receptors (interferon-α/β receptor 1 (IFNAR1)), downstream signaling molecules (Janus Kinase (JAK)) and cytokine-induced transcription factors (Signal transducer and activator of transcription (STAT) 1, STAT3, STAT4)^7^. Also, the activation of Toll-Like Receptors (TLRs) is essential for innate immunity in recognizing microbial components that can trigger release of pro-inflammatory mediators^8^. Thus, there is a plethora of cytokines present during UC inflammation, including TNF, IFNs, IL1β, IL22, and IL17^9^. TNF and IFNγ are both known regulators of epithelial responses eliciting mucosal damage or repair, with dose- and receptor-dependent outcomes (recently reviewed in^10^). IL1β levels are increased in the colonic lamina propria during UC^11,12^, and is associated with neutrophil invasion^13^, Th17 responses and barrier disintegration^14^. Th17 cells produce IL22^15^, which has anti-inflammatory properties and support epithelial repair in rodent models^16^, and IL17, which has both pro- and anti-inflammatory effects in UC^17–19^. Similarly, IFNλ has been shown to induce epithelial proliferation and wound healing but also to impair barrier function through inhibition of e-cadherin and zona occludens-1^20^.

However, the initiating factor of barrier dysfunction remains unresolved. Understanding the epithelial contribution to UC has been challenging due to limitations in model systems that accurately reflect human colonic physiology^21^. Traditional in vitro models often lack the complex architecture and cellular diversity of the human intestinal epithelium, limiting their translational value^22^. In 2009, Sato et al.^23^, introduced intestinal epithelial organoids (IEOs), a groundbreaking 3D model system that allows for prolonged culturing of mini organs (organoids), opening numerous possibilities for research and application. IEOs are generated through the isolation and culturing of stem cells from intestinal biopsy specimens and closely mimic epithelial architecture and function^23^, providing a physiologically relevant platform for studying UC epithelial biology in vitro^24,25^. IEOs preserve the donor’s genetic background^26^, enabling the investigation of patient-specific responses and advancing the potential of precision medicine^22^. A fundamental question is to what extent IEOs recapitulate the intestinal epithelial compartment^24^. For IEOs to be used as a translational medicine tool in UC, we need increased knowledge of how to mimic UC inflammation in vitro. In the present study, we utilized laser microdissected epithelial transcriptomes from paired uninflamed and inflamed colonic epithelium and colonic IEOs established from uninflamed biopsies from the same patients and stimulated with UC-relevant cytokines to investigate the following research questions: What are the key transcriptional programs and biological pathways activated in human IEOs following exposure to UC-associated inflammatory cytokines? To what extent do cytokine-induced transcriptional responses in IEOs recapitulate the epithelial inflammatory signatures observed in UC patients? Which biological processes and molecular pathways demonstrate consistent correlation patterns between the in vivo and in vitro conditions? The answers to these questions could provide insight into how IEOs can be used as a translational model system in IBD precision medicine.

## Methods

**Fig. 1:**
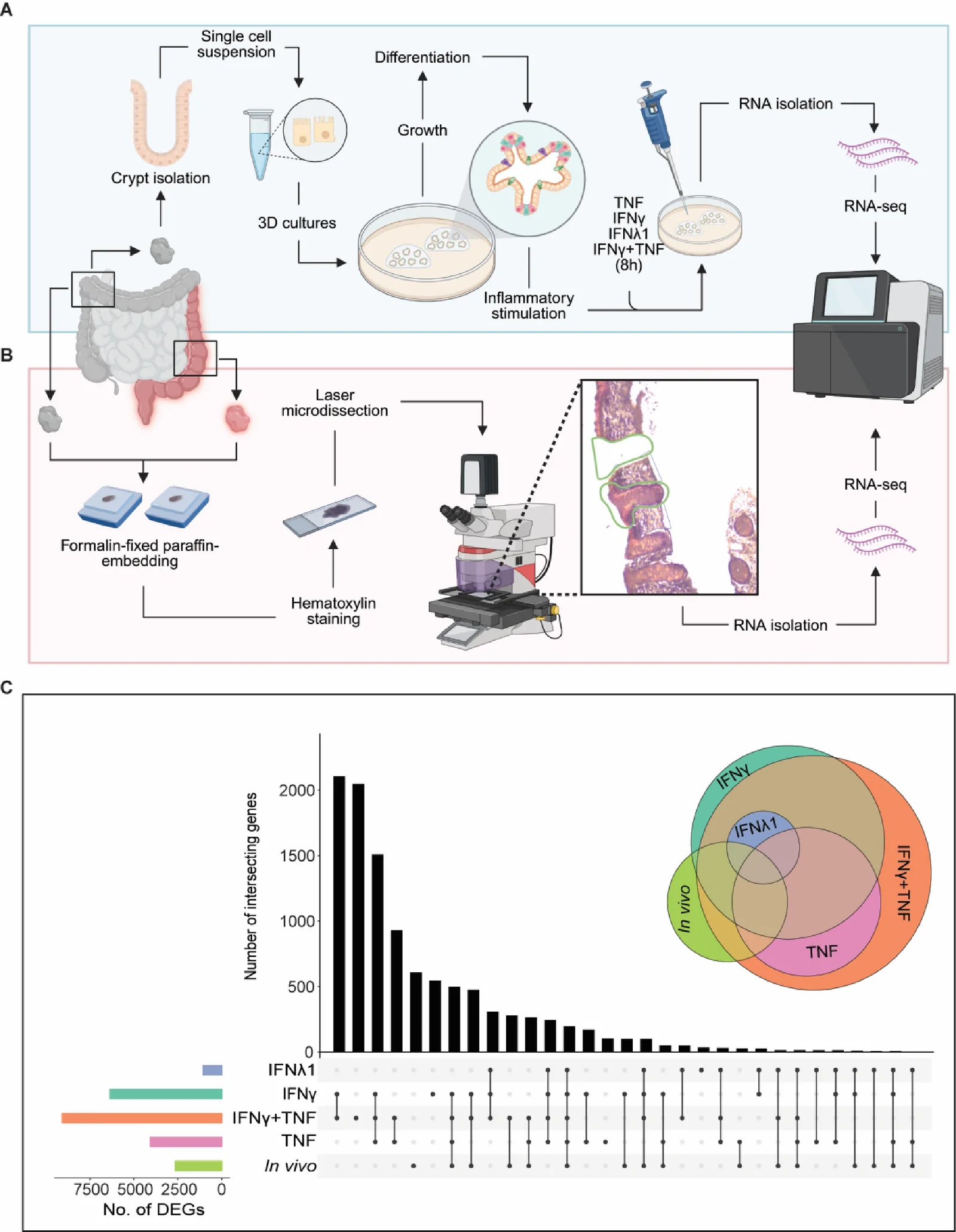
Experimental overview and intersection patterns of differentially expressed genes in vitro and in vivo. Experimental design for collection of human biological material. (**A**) Patient biopsies from the uninflamed right colonic flexure were used for establishment of colonic intestinal epithelial organoids (IEOs). Differentiated IEOs were stimulated with TNF, IFNγ, IFNλ1, or a combination of TNF + IFNγ (8 h) to induce an inflammatory response. mRNA was sequenced for characterization of the difference in inflammatory response between the cytokines. (**B**) Patient biopsies from the uninflamed right flexure and the inflamed descending colon were formalin-fixed and paraffin-embedded into separate blocks, sectioned, hematoxylin-stained and the epithelial compartments isolated using the Leica LMD7 laser microdissection microscope. Total RNA was sequenced for characterization of differences in inflammatory status. (**C**) UpSet and Venn diagram plots depicting the intersection patterns of differentially expressed genes (DEGs) between microdissected samples (in vivo; inflamed epithelium vs. uninflamed epithelium) and IEOs stimulated with the different cytokines IFNγ, IFNλ1, IFNγ + TNF, and TNF (in vitro; cytokine-stimulated vs. unstimulated). Horizontal colored bars indicate the total number of DEGs per condition. Vertical black bars represent the size of intersecting DEG sets, with black dots and connecting lines below indicating contributing condition. The different conditions have designated colors.

Experimental overview is presented in Fig. 1A, B.

### Patient cohort

Patient material was acquired from UC patients with active disease (endoscopic Mayo 2/3) during colonoscopy at St. Olav’s University Hospital, Trondheim, Norway (Table 1). Inflamed (*n* = 12) and uninflamed (*n* = 10) biopsies were simultaneously acquired from the same UC patients (*n* = 12) and formalin-fixed and paraffin embedded (FFPE) for laser microdissection. Uninflamed biopsies (4–5 per donor) for IEO development were collected from the same patient cohort at 12 months follow-up and stored for 24–36 months in liquid nitrogen (*n* = 7). Five patients gave biopsies for both laser microdissection and IEO establishment. Uninflamed biopsies for laser microdissection and IEO development were acquired from the ascending colon while inflamed biopsies were acquired from an inflamed colonic segment. The study was approved by the Central Norway Regional Committee for Medical and Health Research Ethics, Norway (reference number 26798). All patients gave written informed consent, and the experimental methods were performed in accordance with the Declaration of Helsinki.

### Laser microdissection

Total RNA sequencing data of inflamed and uninflamed epithelium in vivo were acquired from an affiliated dataset (GSE288517) and reanalyzed for the purpose of this study. Briefly, the epithelium from paired inflamed (left colon) and uninflamed (right flexure) FFPE biopsies from 12 UC patients (Table 1) were isolated using the Leica LMD7 laser microdissection microscope system (Leica, Wetzlar, Germany). FFPE biopsies were cut into 8 μm sections, mounted on specialized glass slides with a 2.0 μm polyethylene naphthalate (PEN) membrane (#11505189, Leica), and deparaffinized, hematoxylin stained and dehydrated prior to microdissection using a 5x objective lens and laser settings: power 30, aperture 3, speed 5, middlepulse count 3, final pulse 0, head current 100%, pulse frequency 1400. Per biopsy, an average area, including both surface and crypt epithelium, of 862,356 µm2 was collected into microcentrifuge tubes, added 150 µL of PKD digestion buffer (#73505, Qiagen, Hilden, Germany), vortexed, and centrifuged, and stored at -80 °C and until sequencing within a 3-month time frame.

**Table 1:** Donor characteristics.

ID	Disease	Age	Sex	Biopsies for LMD				Biopsies for IEOs				
				5-ASA	GCS	Aza	LMD	IEOs	Passage	anti-TNF^a^	5-ASA	Aza
D05	UC	29	M	Yes	Yes	No	Yes	No	NA	NA	NA	NA
D16	UC	27	F	Yes	No	Yes	Yes	Yes	6	Yes	Yes	No
D21	UC	53	M	Yes	Yes	No	Yes	No	NA	NA	NA	NA
D32	UC	31	F	Yes	No	No	Yes	No	NA	NA	NA	NA
D36	UC	24	F	Yes	No	No	Yes	No	NA	NA	NA	NA
D37	UC	38	F	Yes	No	No	Yes	No	NA	NA	NA	NA
D40	UC	26	M	Yes	Yes	No	Yes	Yes	7	Yes	No	No
D44	UC	25	M	Yes	Yes	Yes	Yes	No	NA	NA	NA	NA
D48	UC	52	F	Yes	Yes	No	No	Yes	9	Yes	No	No
D49	UC	45	M	Yes	Yes	No	No	Yes	5	Yes	Yes	No
D52	UC	44	F	Yes	No	No	Yes	Yes	7	Yes	Yes	No
D57	UC	22	M	Yes	No	Yes	Yes	Yes	7	Yes	Yes	Yes
D65	UC	60	M	Yes	Yes	No	Yes	Yes	11	Yes	Yes	No
D3_65*	UC	18	M	Yes	Yes	Yes	No	Yes	7–9	NA	NA	NA

D – Donor. IEOs – intestinal epithelial organoids. 5-ASA – 5-amonisalisylic acid. Aza – Azathioprine. GCS – Glucocorticoid steroids. LMD – Laser microdissection. NA – Not available. ^a^Anti-TNF was either adalimumab or infliximab. ^*^IEOs (3 independent experiments, each with 4 technical replicates) from this donor were stimulated with a cocktail of cytokines for 24 h before bulk RNA sequencing, as described in the method section.

### Establishment and culturing of intestinal epithelial organoids

The following protocol description for establishing and maintaining IEOs is adapted from Gopalakrishnan et al.^27^, which is based on Mahe et al.^28^: IEOs were derived from 4 to 5 uninflamed biopsies from the right flexure per patient. Immediately after collection, the biopsies were maintained in a cryoprotective solution of fetal calf serum (FCS) and 7.5% dimethyl sulfoxide (DMSO) within a liquid nitrogen tank for long-term preservation. At the outset of organoid establishment, biopsies were carefully thawed and rinsed using 1 mL of FCS and 7.5% DMSO. During subsequent steps, biopsies were consistently kept on ice to minimize cellular stress. The thawed biopsies were then washed with Dulbecco’s phosphate-buffered saline (DPBS, #D8537, Sigma-Aldrich, St. Louis, MO, USA) and transferred to a silicon-bottomed petri dish (Corning, Corning, NY, USA) under a light microscope (Nikon SMZ-2B, Nikon, Japan), where each biopsy was pinned down with the luminal side facing up, and the mucus layer gently removed. The biopsies were washed with a chelation buffer and incubated in an ethylenediaminetetraacetic acid (#15575-038, Sigma-Aldrich)-enriched chelation buffer for 35 min on an orbital shaker to facilitate the release of crypts by gentle scraping. In cases where crypts were not readily released, an additional 15-minute incubation was done. The crypts were washed by centrifugation in 5 + 10 min at 200 rpm, 4 °C. For organoid formation, the pellet was resuspended in 100–200µL of Matrigel (#3018001, Corning) and seeded onto a pre-warmed 24-well cell culture plate. After a settling period of 20 min, 500 µL complete growth media were added and changed every second to third day, containing Y-27,632 (#HY-10583, MedChem Express) the first two days to prevent dissociation-induced apoptosis (anoikis). We expanded the cells at 5% oxygen, and conducted experiments at 2% oxygen since stem cells and differentiated epithelial cells are adapted to slightly different levels of physiological hypoxia^29,30^.

### In vitro experiments with intestinal epithelial organoids

For experimental assays, single cells dissociated from IEOs were resuspended in Matrigel from the same lot (7.7 mg/mL protein) at a concentration of 9000 cells/50µL and plated on pre-warmed 24-well plates (2 × 25µL domes/well). The organoids were cultured as described above until 9 days post plating when the IEOs were cultured in differentiation media containing 5% Wingless-related integration site (WNT) (Wnt3A conditioned medium, #CRL-2647, ATCC) and 4.3 µg/mL of DAPT (Notch signaling inhibitor)^27^, with additional media changes occurring on days 10 and 13. To reproduce the inflammatory environment of active UC, we stimulated the IEOs on day 14 with pro- and anti-inflammatory cytokines including TNF (100 or 10 ng/mL) (#300–01 A), IFNγ (20 or 2 ng/mL) (#300-02BC, ), IFNλ1 (20 ng/mL) (#300–02 L), and IFNγ + TNF (10 ng/mL + 50 ng/mL), all from PeproTech (Rocky Hill, NJ, USA). After 8 h of cytokine stimulation, the cells were lysed for RNA analyses by washing with pre-warmed DPBS, adding 250 µL ice cold lysis buffer with beta-mercaptoethanol, scraping and sheering through sterile 20-gauge needles ten times to get a homogenous lysate before storing at -80 °C. Each condition had four technical replicates that were combined. Further, in an in-house bulk RNA sequencing dataset (GSE289072), IEOs from a separate UC patient (D3_65, Table 1) were stimulated with a cocktail of cytokines for 24 h (3 independent experiments, each with 4 technical replicates). The cocktail included the TLR3 ligand Polyinosinic: polycytidylic acid (Poly(I: C)) (0.63 µg/mL) (#tlrl-pic, Invitrogen, Waltham, MA, USA) and 5 cytokines with the relatively low concentration of 3.13 ng/mL: TNF (#300–01 A), IFNγ (#300-02BC), IL1β (#200-01B), IL17 (#17502001), IL22 (#200 − 22), all from PeproTech.

### RNA isolation and sequencing

#### Laser microdissected material

In vivo epithelial transcriptomes were prepared as described (GSE288517). Briefly, total RNA was extracted using the RNeasy^®^ FFPE Kit (#73504, Qiagen) following the manufacturer’s protocol, with the additional step of incubating the lysate at room temperature for 10 min prior to elution. RNA was quantified using the Qubit^®^ RNA HS Assay Kit (#Q32855, Invitrogen), and RNA integrity evaluated on an Agilent 2100 Bioanalyzer using the RNA 6000 Pico Kit (Reagent #5067 − 1513, Ladder #5067 − 1535, Agilent Technologies, Santa Clara, CA, USA). RNA sequencing libraries were constructed using the Illumina Stranded Total RNA Prep with Ribo-Zero kit (#20040529, Illumina, San Diego, CA, USA) utilizing all RNA available (6 to 55 ng per sample). Ribosomal RNA was depleted prior to fragmentation (2 minutes at 94 °C), first-strand cDNA synthesis conducted with random hexamers and an Actinomycin D-supplemented First Strand Synthesis Mix, and deoxyuridine triphosphate (dUTP) incorporated during the second-strand synthesis step. Library preparation continued with 3’ adenylation, adapter ligation, and PCR amplification (15 cycles) using Illumina^®^ RNA UD Indexes Set C (#20091659, Illumina). Libraries were purified with the Mag-Bind TotalPure NGS Kit (#M1378-01, Omega Bio-Tek, Norcross, GA, USA), quantified using the Collibri™ Library Quantification Kit (#A38524500, Invitrogen), and validated on an Agilent TapeStation 4200 (Model G2991BA, Agilent Technologies). Fragment sizes were confirmed to range between 200 and 500 bp, with an average peak of ~ 280 bp. Sequencing was carried out on a NovaSeq6000 platform using an S2 flowcell (#20028316, Illumina) for 138 cycles with a 59-10-10–59 bp read configuration, following the manufacturer’s instructions.

#### Intestinal epithelial organoid material

Total RNA from IEOs was extracted using the RNeasy mini kit (#74106, Qiagen) and concentration and quality was evaluated with NanoDrop Spectrophotometer (Thermo Scientific, Massachusetts, United States). RNA sequencing libraries were prepared using the Illumina Stranded mRNA Prep (Ligation) Kit (#20040534, Illumina), following the manufacturer’s protocol. Briefly, 300 ng total RNA was used as the starting material. First, mRNA was isolated from the total RNA using poly-T oligo-attached magnetic beads, followed by random fragmentation at 94 °C for 8 minutes. First-strand cDNA synthesis was performed at 42 °C for 15 minutes using random hexamer oligonucleotides and Actinomycin D, enhancing strand specificity by supporting RNA-dependent synthesis while preventing spurious DNA-dependent synthesis. Next, second-strand cDNA synthesis was conducted at 16°C for 1 hour, during which the RNA template was removed, and a complementary strand was synthesized to create blunt-ended, double-stranded cDNA fragments. To achieve strand specificity, dUTP was incorporated instead of deoxythymidine triphosphate (dTTP) during this step, quenching the second strand during amplification. Subsequently, 3’ end adenylation was performed at 37 °C for 30 min, followed by the ligation of anchors and Illumina dual index adapter oligonucleotides at 30 °C for 10 min. Library fragments underwent cleanup using Mag-Bind TotalPure NGS Kit (Omega Bio-Tek, Cat. No. M1378-01) and were enriched through 11 cycles of PCR. The final libraries were purified with Mag-Bind TotalPure NGS Kit (Omega Bio-Tek, Cat. No. M1378-01), quantitated using the Collibri™ Library Quantification Kit (Thermo Fisher Scientific, Cat. No. A38524500) and validated with the Agilent High Sensitivity DNA Kit on a Bioanalyzer (5067 − 4626, Agilent Technologies). The size of the DNA fragments was determined to range from approximately 200 to 600 bp, with a peak around 300 bp. The libraries were then pooled and normalized to 1.0 nM (final loading concentration of 200 pM) before clustering on a NovaSeq 6000 S2 flow cell (Illumina). Paired-end sequencing was performed on the NovaSeq 6000 instrument for 138 cycles with a 59-10-10–59 bp read configuration, following the manufacturer’s instructions.

### Post sequencing processing

FASTQ files were generated with bcl2fastq2 Conversion Software v2.20.0.422 (Illumina), the quality assessed using FastQC (v0.11.9), followed by filtering and trimming with fastp (v0.20.1). Trimmed sequences were then aligned to the reference genome using STAR aligner (v2.7.9a), and quality metrics were extracted with Picard’s CollectRNASeqMetrics tool (v2.21.5). Transcript quantification was performed using quasi-alignment with Salmon (v1.7.0) against the GRCh38 transcriptome reference sequences. Transcript counts were imported into R statistical software and aggregated to gene-level counts using the tximport package (v1.20.0). Gene counts were normalized, and differential expression analysis was conducted using the DESeq2 Bioconductor package^31^. For multivariate analyses, data underwent variance-stabilizing transformation. To visualize intra-gene sample differences, we utilized normalized counts based on the median of ratios method.

### Immunoblotting

Proteins were extracted from the IEOs by lysing on ice using lysis buffer containing 50 mM Tris-HCl pH 7.5, 150 mM NaCl, 5 mM EDTA (#15575-038, Invitrogen), 1% NP-40 (#492018, Sigma-Aldrich), 1 mM dithiothreitol, 1x Complete^®^ EDTA-free protease inhibitor (#11873580001, Sigma-Aldrich), and 1x phosphatase inhibitor cocktail II (#P5726, Sigma-Aldrich) and III (#P0044, Sigma-Aldrich), respectively for 2 h. Lysates were clarified centrifugation at 13,000 × g for 15 min at 4 °C, and protein concentration was measured using the Pierce BCA protein assay (#23225, Thermo Fisher Scientific). Protein lysates were then denatured in 1 × NuPage lithium dodecyl sulfate sample buffer (#NP0007, Invitrogen) supplemented with 1 mM dithiothreitol for 10 min at 70 °C before they were separated on 4–12% NuPage Bis-Tris gels (#NP0321BOX, Invitrogen) and electroblotted onto nitrocellulose membranes (0.2 μm, #1704158, Bio-Rad) using the Trans-Blot Turbo Transfer System (Bio-Rad). Overnight dried membranes were blocked using Rockland Blocking Buffer for Fluorescent Western Blotting (#MB-070, Rockland) for 1 h at room temperature before indicated primary antibodies incubation overnight at 4 °C. The following day, membranes were washed 3 × 10 min in TBS-Tween (0.05%) (TBST) prior to incubation with indicated Dylight-conjugated secondary antibodies for 1 h and again washed before visualization. The following primary antibodies and dilutions were used: Endoplasmic reticulum aminopeptidase 2 (ERAP2) goat monoclonal antibody (R&D Systems, 1:2000, #AF3830), Interferon regulatory factor 1 (IRF1) rabbit monoclonal antibody (Abcam, 1:1000, #Ab186384) and Glyceraldehyde-3-phosphate dehydrogenase (GAPDH) (D16H11) rabbit monoclonal antibody (Cell Signaling, 1:5000, #5174). Secondary antibodies used were Goat anti-Rabbit DyLight™ 800 (1:5000, #SA5-35571) and Donkey anti-Goat DyLight™ 647 (1:1000, #A-21447) (both from Invitrogen). Images were obtained with LI-COR Odyssey Imager and analyzed using Image Studio Software (LI‐COR Biosciences). Total protein levels were normalized to GAPDH expression and presented as fold change (Supplementary File 1).

### Immunostaining

Colonoids collected for immunostaining were resuspended in 50 m L Richard- Allan Scientific™ HistoGel ™ Specimen Processing Gel (#HG-4000-012, Thermo Fisher Scientific, Waltham, MA), then fixed in 10% buffered formalin for 24 h before they were embedded in paraffin. Section  (4 μm) of FFPE colonic tissue or IEOs were baked at 60 °C (30 min) prior to de-paraffinization in Neo-Clear^®^ and rehydration through an ethanol gradient and dH_2_O. Sections were quenched for endogenous peroxidase in 3% H_2_O_2_ (10 min) (for immunohistochemistry) prior to heat-induced antigen retrieval (15 min, Tris-EDTA buffer, pH 9) and blocking (30 min, room temperature) by Normal Goat Serum Blocking Solution (Vector Laboratories, #S-1012-50) (immunohistochemistry) or 3% BSA-TBST (immunofluorescence). In both procedures, TBST was used for washing steps (3 × 5 min) between primary antibody incubation, secondary antibody incubation, and nuclear staining. Primary antibody incubations were done overnight at 4 °C and include IRF1 (Abcam, 1:1000, #ab186384), dual oxidase 2 (DUOX2) (Novus Biologicals, 1:300, #NB110-61576), and inducible nitric oxide synthase (iNOS) (R&D Systems, 1:100, #MAB9502).

### Immunohistochemistry

Visualization was performed using the Rabbit/Mouse Dako REAL EnVision Detection System (K500711-2, Dako, Glostrup, Denmark) following the manufacturer’s instructions. Hematoxylin was used for nuclear staining (20 s).

### Immunofluorescence

Secondary antibody incubations (1:1000) were carried out at room temperature (1 h), using Alexa Fluor™ 488 (A11008, Invitrogen) for detection of DUOX2 and Alexa Fluor™ 555 (Vector Laboratories A21422, Invitrogen) for detection iNOS. 4′,6-diamidino-2-phenylindole (DAPI) (Vector Laboratories 62248, Thermo Scientific™) was used for nuclear staining (1:1000, 5 min). Sections were mounted in ProLong™ Gold Antifade Mountant (Vector Laboratories P36934, Invitrogen™) and imaged on a LSM Airyscan (ZEISS) using the ZEN Microscopy Software (blue edition).

### Bioinformatics and statistical analyses

Statistical analyses were performed using R software (version 4.4)^32^. Differential gene expression analysis resulted in multiple gene lists that were analyzed for intersecting patterns. Visualization of gene set intersections was performed using the UpsetR and Euler packages^33,34^ and ggplot2 was employed for additional data visualization. Over-representation enrichment analyses were conducted with Metacore (Clarivate, Philadelphia, PA, USA) and supported by DAVID^35,36^ and gprofiler2^37^. Input gene sets were defined using differential expression thresholds (log2 fold change (FC) > 0.3 and < -0.3, and adjusted p-value < 0.05, baseMean > 20). For targeted analysis of pathway-specific gene expression, pathways and genes were selected based on published literature on UC epithelial inflammation and from enriched process networks identified by over-representation analysis. For correlation analyses between in vivo and in vitro responses, we analyzed patient-matched samples from individual patients (*n* = 5) who provided both laser microdissected inflamed colonic epithelium and IFNγ + TNF-stimulated IEO samples. Gene-specific correlations were calculated using Pearson’s correlation coefficient, with p-values adjusted for multiple comparisons using the Benjamini-Hochberg procedure. Data normality was assessed using the Shapiro-Wilk test, and statistical comparison between paired (uninflamed/inflamed and unstimulated/stimulated) samples were conducted using paired t-tests.

### Declaration of generative AI and AI-assisted technologies

During the preparation of this work, the author(s) used Claude.ai (Anthropic, San Francisco, CA, USA) in order to edit the text since English is not the authors native language and provide assistance writing R syntax. The Chat & Ask AI language model (GPT-4o) (Codeway, Istanbul, Turkey) was also employed for R programming tasks. After using these tools or services, the author(s) reviewed and edited the content as needed and take(s) full responsibility for the content of the publication.

## Results

### Inflamed UC epithelial tissue and cytokine-stimulated IEOs share signature processes

**Fig. 2:**
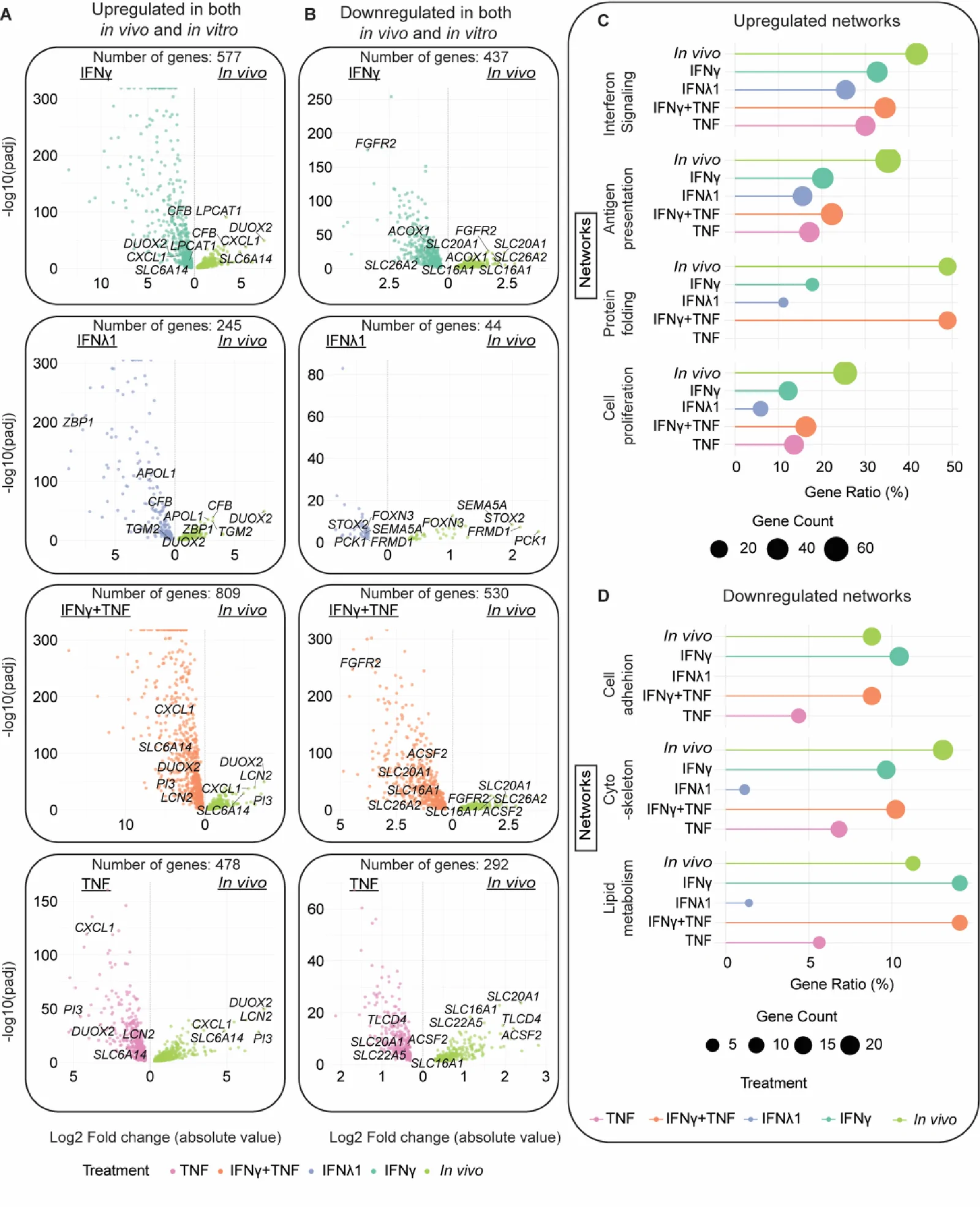
Integration of transcriptional epithelial responses from UC patients and cytokine-stimulated organoids reveal shared programs. Volcano plots displaying the upregulated (**A**) and downregulated (**B**) differentially expressed genes (DEGs) contrasting inflamed vs. uninflamed epithelial samples in vivo with cytokine-stimulated intestinal epithelial organoids (IEOs) in vitro. The different conditions have designated colors. The DEGs with the largest effect size in vivo that are present also in vitro, are highlighted. (**C**,**D**). Gene set enrichment analysis visualized as lollipop plots showing selected top upregulated- (**C**) and downregulated (**D**) process networks. Lollipop length corresponds to the gene ratio (number of DEGs /total genes in pathway) and dot size the number of DEGs involved in each network. The different conditions have designated colors. (**C**,**D**) represent enrichment of overlapping genes between in vivo and in vitro. See also Supplementary File 2.

Since IEOs are utilized as preclinical models to understand pathophysiological mechanisms, it is vital to understand which processes are activated with a given cytokine and whether these processes are relevant for UC. Therefore, we performed differential expression analysis on cytokine-stimulated (IFNγ, IFNλ1, TNF, and IFNγ + TNF) IEOs from UC patients (*n* = 7) and compared the gene lists to differentially expressed genes (DEGs) between paired inflamed (*n* = 12) and uninflamed (*n* = 10) microdissected colonic epithelium from the same patient cohort. Our analysis (log2FC > 0.3 and < -0.3, p-adjusted < 0.05, baseMean > 20) revealed extensive transcriptional changes in both in vivo and in vitro conditions, with 2,654 DEGs between inflamed and uninflamed epithelium and the stimulated IEOs exhibiting distinct transcriptional responses: 6,357 DEGs with IFNγ, 1,069 with IFNλ1, 4,070 with TNF, and 9,067 with IFNγ + TNF compared to unstimulated controls (Fig. 1C, colored bars). Analysis of transcriptional overlap revealed the highest concordance (2,106 DEGs) among IFNγ and IFNγ + TNF (Fig. 1C, black bars and Venn diagram). The in vivo inflamed epithelium signature shared 498 genes with IEOs stimulated with IFNγ, TNF, or IFNγ + TNF (Fig. 1C and 7th black bar), while 196 DEGs were common across all conditions (Fig. 1C and 13th black bar). We also observed that there were some condition-specific responses with 608 DEGs unique to the inflammation in vivo and 546, 35, 103, 2,047 DEGs unique to IFNγ, IFNλ1, TNF and IFNγ + TNF stimulation, respectively. Directional analysis of commonly regulated genes between in vivo and in vitro conditions revealed 1014, 289, 1339, and 770 concordantly regulated genes across the IFNγ, IFNλ1, IFNγ + TNF, and TNF stimulations (Fig. 2A, B). Notably, in vitro stimulation generally yielded larger effect sizes with more variance than inflammation in vivo. Key inflammatory mediators such as *DUOX2*,* CXCL1*, and *SLC6A14* were consistently upregulated (Fig. 2A), and *SLC16A1*,* SLC20A1* were consistently downregulated (Fig. 2B) in both in vivo inflamed epithelium and cytokine-stimulated in vitro IEOs, except under IFNλ1 stimulation. *LCN2*, which was upregulated *in vivo*, was only induced by TNF in vitro.

Over-representation enrichment analysis identified shared biological processes between in vivo and in vitro conditions which are represented as gene ratio (%) (Supplementary File 2). The most enriched processes such as interferon signaling, antigen presentation, protein folding, and cell proliferation were consistently upregulated across all cytokine stimulated conditions, except for TNF, where protein folding was absent from the top 50 identified process networks. (Fig. 2C). Although the cytokine conditions displayed substantial similarities, there were some relative differences; IFNγ predominantly drove interferon signaling and antigen presentation pathways, TNF regulated genes associated with cell proliferation, while the combination of IFNγ and TNF enhanced protein folding responses. Processes related to cell adhesion, cytoskeleton organization, and lipid metabolism showed significant downregulation both in vivo and in vitro, primarily driven by IFNγ stimulation in vitro (Fig. 2D).

### Cytokine concentrations and stimuli combinations affect gene expression

**Fig. 3:**
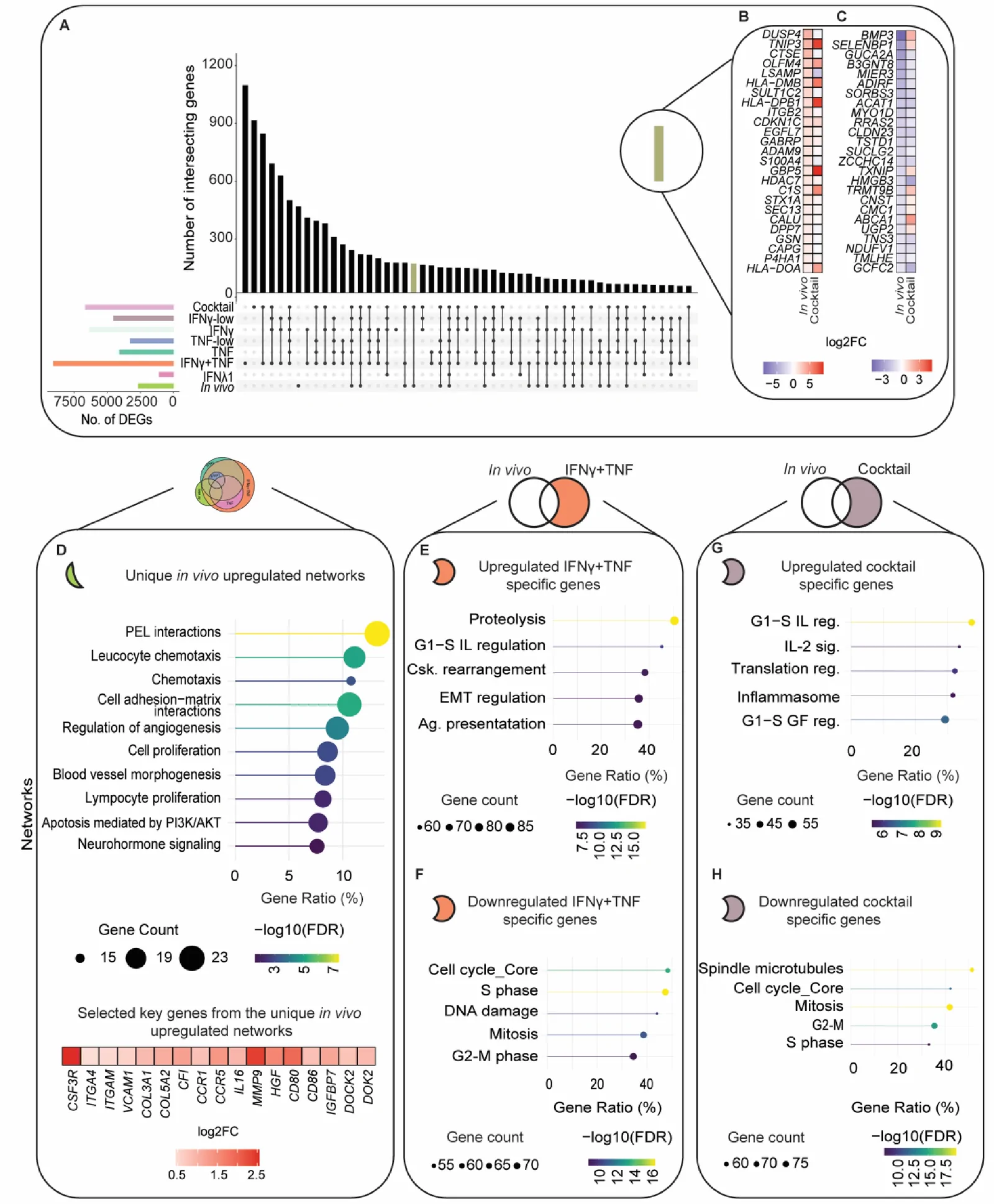
Low concentrations of pro- and anti-inflammatory cytokine cocktail can also mimic UC-like inflammation in IEOs. (**A**) UpSet plot showing intersections of differentially expressed genes (DEGs) between laser microdissected samples (in vivo; inflamed vs. uninflamed) and intestinal epithelial organoids (IEOs) (in vitro; stimulated vs. unstimulated). IEOs were treated with IFNγ, IFNλ1, IFNγ + TNF, TNF, or a low-dose inflammatory cocktail (3.13 ng/mL each of TNF, IFNγ, IL1β, IL17, IL-22, and 0.63 µg/mL of Poly(I:C)). Horizontal colored bars indicate the total number of DEGs per condition. Vertical black bars represent the size of intersecting DEG sets, with black dots and connecting lines below indicating contributing conditions. (**B**,**C**) Heatmap of top 25 DEGs uniquely overlapping between cocktail-stimulated IEOs and the inflamed in vivo epithelium. Color intensity represents log2 fold change with red/blue indicating the up/down-regulation compared to the respective controls (in vivo: uninflamed; in vitro: unstimulated). (**D**–**H**) Gene set enrichment analysis visualized as lollipop plots. Panels (**D**,**E**,**G**) show up-regulated pathways; (**F**,**H**) show downregulated pathways. Lollipop length corresponds to the gene ratio (number of DEGs /total genes in pathway), and dot size the number of genes involved in each network. The color gradient corresponds to significance level (-log10(false discovery rate (FDR))). (**D**) Enrichment analysis of upregulated DEGs uniquely expressed in vivo together with a heatmap highlighting a subset of genes involved in the networks Regulation of angiogenesis, Chemotaxis and lymphocyte proliferation. The color intensity represents log2 fold change. (**E**–**H**) represents processes regulated in vitro from DEGs that are not present in vivo; (**E**,**F**) when IEOs are stimulated with IFNγ + TNF and (**G**) and (**H**) when IEOs are stimulated with the inflammatory cocktail. PEL – Platelet-endothelium-leukocyte. Csk. – Cytoskeleton. Sig. – Signaling. Ag. – Antigen. IL – Interleukin. GF – Growth factor. EMT – Epithelial-to-mesenchymal transition. Lym. – Lymphocyte. Reg. – Regulation.

TNF and IFNγ are central regulators of epithelial barrier integrity, but their elicited effects on the epithelium are regulated by the duration of the stimuli, the concentration and the receptor they engage^10^. Further, numerous cytokines are present during UC inflammation^38^. We therefore extended our analysis to include IEOs stimulated with reduced IFNγ and TNF concentrations as well as a pro-inflammatory cocktail of cytokines and a TLR3 ligand at reduced concentrations (TNF, IFNγ, IL1β, IL17 and IL22 + Poly(I:C)). The cocktail results were acquired from an independent IEO dataset (GSE289072). Although direct comparisons between the two experiments are limited due to different stimulation timing and donor populations, upset plot analysis revealed that cocktail stimulation induced a comparable number of DEGs (6643) compared to IFNγ + TNF (9067) (Fig. 3A, colored bars), and 480 DEGs were commonly regulated across all cytokine-stimulated conditions except IFNλ1 (Figs. 3A and 7th black bar). We also found that stimulation with 10-fold reduced concentrations of IFNγ or TNF resulted in almost the same number of DEGs (4517 vs. 6357 and 3264 vs. 4070, respectively). Notably, the transcriptional changes following stimulation with low vs. high concentrations of IFNγ and TNF showed no significant differences for TNF and only 1058 significant DEGs for IFNγ with similar responses but with reduced magnitude (data not shown).

Among the tested stimulation conditions, only the cocktail, IFNγ + TNF and IFNγ stimulations (high and low concentrations) induced their own distinct sets of DEGs with the IFNγ + TNF stimulation showing the highest number of 1076 genes (Fig. 3A, 1st black bar). Furthermore, there were only 151 genes which were uniquely co-regulated between in vivo and cocktail-stimulated IEOs independent of the other cytokine stimulations (Fig. 3B and C). This observation indicates that the coordinated action of multiple inflammatory mediators at reduced concentrations (cocktail) similarly can reproduce the inflammatory signature achieved by higher doses of IFNγ + TNF, but without providing substantial additional insight to the in vivo condition compared to using more defined stimulations.

For a translational model system, it is equally important to identify which in vivo processes that are not replicated in vitro (and vice versa). Our data (Figs. 2 and 3A) demonstrate that IFNγ + TNF-stimulated IEOs effectively model many epithelial inflammatory processes observed in vivo. However, the 448 DEGs uniquely present in vivo were enriched for processes related to intraepithelial immune cells and mesenchymal cells; platelet-endothelium-leucocytes interaction (PEL); leucocyte chemotaxis; cell matrix interactions (Fig. 3D), highlighting the proximity and interactions between epithelial cells and other cell types in vivo that are not present in the IEO model.

Genes specifically upregulated by IFNγ + TNF stimulation were enriched for fundamental cellular process including proteolysis, interleukin-driven regulation of G1-S transition, cytoskeleton rearrangement, extracellular matrix regulation, and antigen presentation (Fig. 3E). Downregulated processes in this condition were primarily related to cell cycle progression and cytoskeleton organization such as S phase, mitosis, DNA double strand breaks repair, and core cell cycle components (Fig. 3F). The pro-inflammatory cocktail induced a similar, yet distinct set of responses. Upregulated processes unique to cocktail include G1-S cell-cycle regulation (both interleukin and growth factor-dependent G1-S transition), translation regulation and immune-related processes such as IL-2 signaling, and inflammasome (Fig. 3G). Downregulated processes in this condition were also predominantly related to cell-cycle progression including G2-M transition, mitosis, core cell-cycle components, S phase, and spindle microtubule organization (Fig. 3H). These findings suggest that both IFNγ + TNF and the cocktail induced cell cycle and protein homeostasis responses in vitro that are not fully reflected in the in vivo inflammatory state.

### Inflammation-associated genes show conserved regulation patterns between UC epithelium and cytokine-stimulated IEO for a core set of genes

**Fig. 4:**
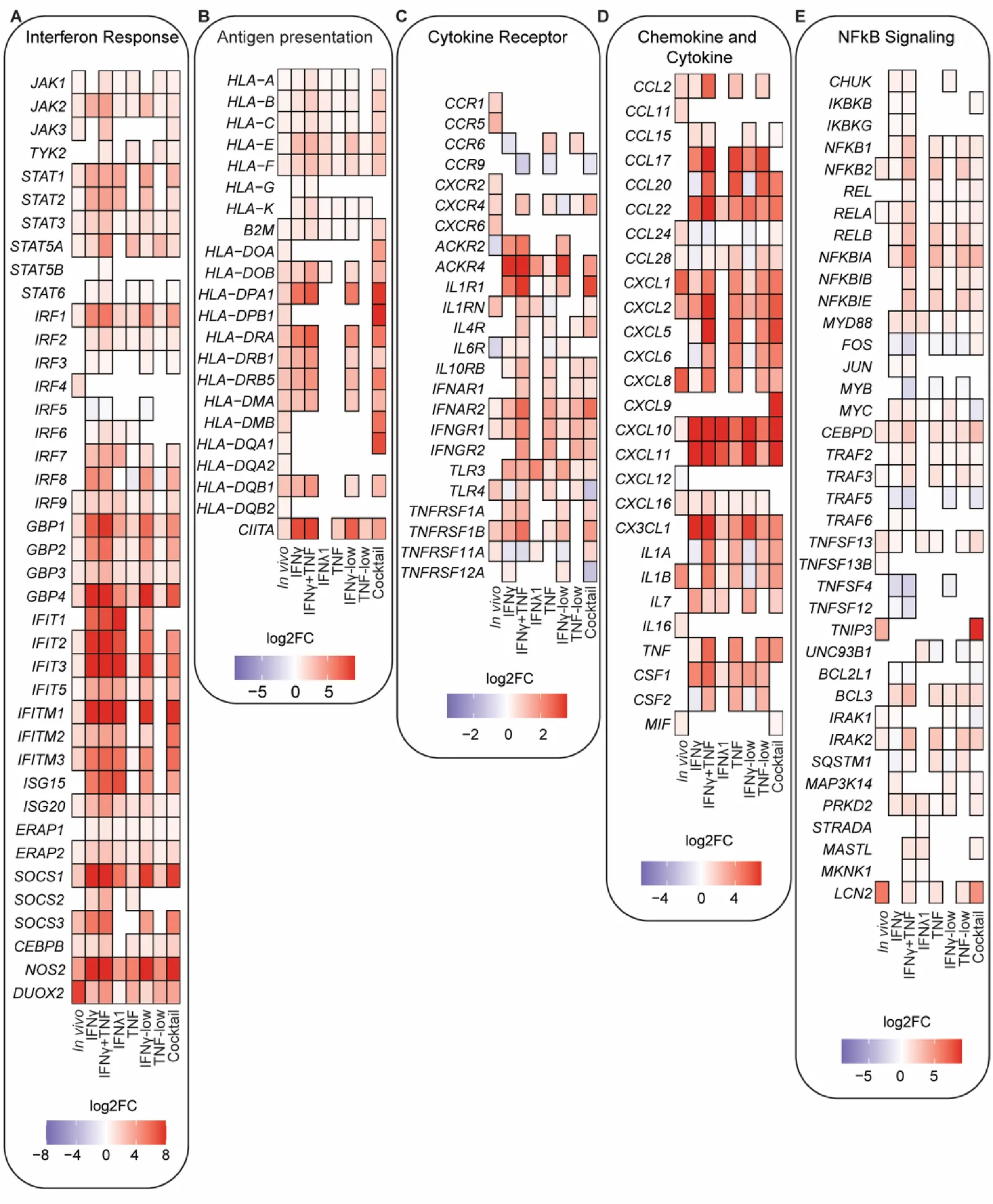
Regulation of genes central to UC-associated processes are reproduced in vitro. (**A**–**E**) Heatmaps showing the significantly differentially expressed genes (DEGs) selected from different enriched process networks shown in Fig. 2C, D. Color intensity represents log2 fold change with red/blue indicating the up/down-regulation compared to the respective controls (in vivo: uninflamed; in vitro: unstimulated). Empty cells are genes not significantly differentially expressed within the given condition. (**A**) Interferon signaling-associated genes, (**B)** Antigen presentation-associated genes, (**C**) Cytokine receptors, (**D**) Chemokines and cytokine, and (**E**) NFκB-associated genes.

To assess the biological relevance of the enriched pathways (Fig. 3), we analyzed expression of key IBD-associated genes involved in important immune signaling pathways^9,39–42^(Fig. 4). Most genes involved in interferon signaling displayed robust and consistent upregulation both in vivo and in vitro (Fig. 4A). Key components in this pathway, including *JAK1*,* JAK2*,* STAT1*,* STAT2*,* STAT3*,* STAT5A* and downstream effectors such as *IRF1*,* IFIT2-5*,* IFITM1-3*, *ERAP2*, *ISG20*,* SOCS1*,* NOS2* and *DUOX2*, showed coordinated activation. While *TYK2* was not detected in vivo, in vitro IEO experiments showed that its expression was induced primarily by TNF.

MHC-related genes exhibited more complex regulation patterns (Fig. 4B). MHC class I genes showed consistent expression across in vivo and in vitro (except for *HLA-G* and *HLA-K*). In contrast, MHC class II genes displayed condition-specific regulation. Genes such as *HLA-DOB*,* HLA-DPA1*,* HLA-DRA*,* HLA-DRB1*,* HLA-DRB5*,* HLA-DMA*, and *HLA-DQB1* were commonly regulated between the in vivo condition and IEOs stimulated with IFNγ, IFNγ-low, IFNγ + TNF or cocktail. However, another set of MHC class II genes such as *HLA-DOA*,* HLA-DPB1*,* HLA-DMB*, and *HLA-DQA1* were exclusively regulated in vivo and by the cocktail (Fig. 3B), highlighting that the cocktail uniquely captured the complexity of more antigen presenting genes in the mucosal epithelium. IFNλ1 only significantly induced transcription of MHC class I related genes and not MHC class II.

The connection between the JAK-STAT signaling transduction and certain chemokines is influenced by cytokine receptor subunits. Analysis of receptor genes expression demonstrated a distinct pattern between in vivo and in vitro conditions (Fig. 4C). The interferon receptor family *IFNAR2* and *IFNGR1* were upregulated both in vivo inflammation and in response to all cytokine stimulations in vitro, except by IFNλ1. In contrast, *IFNAR1* and *IFNGR2* were not significantly regulated in vivo but were upregulated in vitro under TNF-specific stimulation. Thus, TNF stimulation induced IFNα and IFNγ receptor expression, while IFNγ strongly induced TNF receptor expression; *TNFRSF1A* was exclusively regulated in vitro by IFNγ-specific and cocktail stimulations, whereas *TNFRSF1B* additionally was differentially expressed *in vivo* and self-regulated by TNF stimulation. *CCR1*,* CCR5*,* CXCR2* and *CXCR6* were only induced *in vivo*, while *CXCR4* showed specific induction *in vivo* and during TNF-related stimulation. Several other receptors such as *TNFRSF12A*,* ACKR4*,* IL1R1*,* IL4R*,* IL10RB*,* TLR3*,* CCR6*,* and CCR9* were only significantly regulated in vitro.

The chemokine and cytokine genes expression profile demonstrated that only a selected set of inflammatory mediators (*CCL2*,* CCL11*,* CCL24*,* CCL28*,* CXCL1*,* CXCL2*,* CXCL8*,* CXCL10*,* CXCL12*,* CXCL16*,* IL1β*,* IL16* and *MIF*) were significantly regulated in vivo (Fig. 4D). Both IFNγ + TNF and the cocktail stimulation closely resembled the in vivo expression pattern, strongly inducing IFN-responsive chemokine (*CXCL2/10*) while also capturing broader inflammatory responses including *CXCL5/11*,* CXC3CL1*,* IL1β*, *IL7*, *TNF*,* CCL20*, and *CCL22*. IFNγ stimulation alone induced similar number of cytokines compared to IFNγ + TNF, with notable exceptions in downregulation of *CSF2*, *CCL20*, *CCL28*, *CXCL6*,* IL1A*. IFNλ1 stimulation had limited effects, only regulating a specific set of genes: *CCL22*, *CCL28*,* CXCL10*,* CXCL11*, *CXCL16*, *CXC3CL1*, *IL1A*, *IL1β*, *IL7* and *CSF1.* In contrast to the low concentration of TNF, IFNγ-low stimulation downregulated *CSF2*, *CCL20*, *CCL28*, *CXCL6*,* IL1A*,* IL1β.*The genes *IL1A*,* IL1β*,* CSF2*, *CXCL1*, *CXCL5*, *CXCL6*, *CXCL8* and *CCL20* generally displayed TNF-driven regulation, with *IL1A*, *IL1β*, *CSF2*, *CXCL6* and *CCL20* being oppositely downregulated by IFNγ alone. *CXCL9* was induced only by cocktail stimulation, while *CCL11*, *CXCL12* and *IL16* were only regulated in vivo (Fig. 4D).

NFκB is a pivotal inflammatory transcription factor regulating epithelial survival and apoptosis, but has also been shown to have beneficial effects in epithelial cells, playing and important role in maintaining intestinal homeostasis^10^. Our analysis also revealed an interesting pattern in its regulation. In the *in vivo* condition, genes encoding the inhibitor of kappaB kinase (IκK) complex components *CHUK (I*κ*K1)*,* IKBKB (I*κ*K2)* and *IKBKG* (*NEMO*) (thus being activators of NFκB) showed no regulation, though *IRAK1* and *IRAK2* were regulated. This selective regulation through interleukin-1-receptor associated kinases might explain why only certain components of NFκB signaling pathway (*NFKB2*,* RELA*,* CEBPD*,* TRAF3*,* TNFSF13*, *TNFSF13B*, *TNIP3*, *IRAK1*, and *IRAK2*) showed regulation *in vivo*. In contrast, in stimulated IEOs, *CHUK*, *IKBKB* and *IKBKG* genes were activated in response to IFNγ-specific stimulations. Even if key components of the NFκB (*NFKB1*, *NFKB2*, *REL*, *RELA*, *RELB*) and its inhibitors NFκB inhibitor alpha (*NFKBIA*), beta (*NFKBIB*) and epsilon (*NFKBIE*) to some extents were regulated by IFNγ, they were mainly TNF-driven (Fig. 4E). As described in Fig. 2, *LCN2* displayed TNF-specific regulations.

In summary, detailed examination of pathway-specific gene expression patterns revealed that core inflammatory processes, particularly interferon signaling and antigen presentation pathways, show conserved regulation in UC tissue and cytokine-stimulated organoids, though with distinct differences in magnitude and complexity. Generally, in vitro cytokine stimulation induced stronger transcriptional responses compared to in vivo epithelial inflammation, with notable exceptions being *DUOX2* and *LCN2*. These expression patterns further support our pathway analysis findings while highlighting both the similarities and limitations of cytokine-stimulated IEOs in modelling UC inflammation.

### Paired in vivo and in vitro transcriptomes of UC-related genes are reflected at protein level

**Fig. 5:**
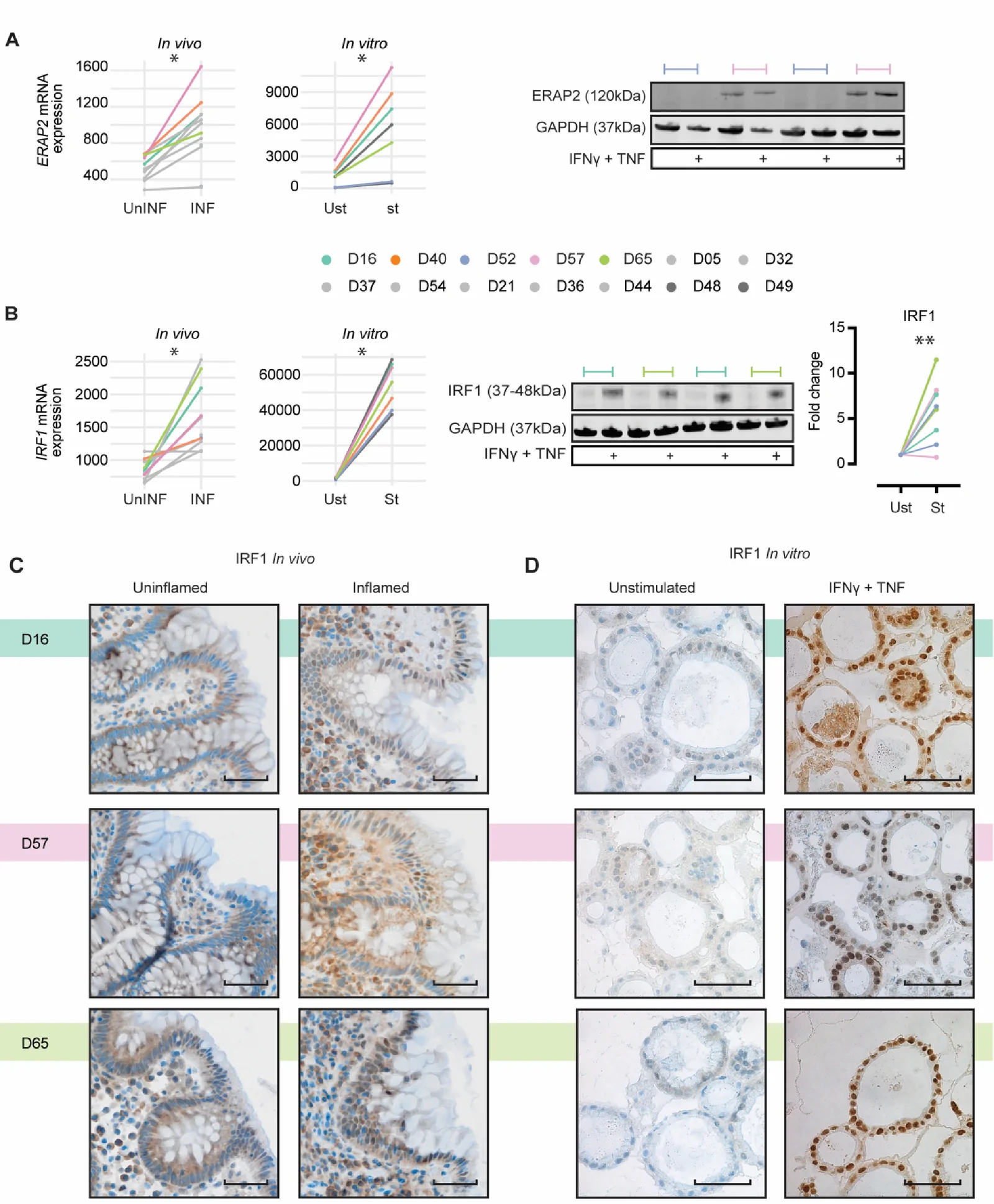
Protein expression of ERAP2 and IRF1 correlates with mRNA expression of *ERAP2* and *IRF1*. Paired comparison of (**A**) *ERAP2* and (**B**) *IRF1* expression levels between uninflamed vs. inflamed epithelium in vivo, and unstimulated vs. IFNγ + TNF-stimulated IEOs in vitro. Each line represents data from the same donor, illustrating within-patient changes. Colored lines represent overlapping in vivo and in vitro donors (*n* = 5), while light grey are in vivo only donors (*n* = 7), and dark grey are in vitro only donors (*n* = 2). Significance values were obtained from differential expression analysis dataset processed by DESeq2 Bioconductor package (* *p* < 0.05). Western blot analysis of (**A**) ERAP2 protein levels in IEOs from D52 and D57 and (**B**) IRF1 protein levels in IEOs from D16 and D65, together with a paired comparison of IRF1 fold changes in western blot analysis (*n* = 8, 4 donors with independent experiments). Statistical significance of fold change was assessed using paired t-test (** *p* < 0.01). Representative immunohistochemical images from D16, D57, and D65 showing increased nuclear IRF1 staining (brown) in (**C**) inflamed vs. uninflamed colonic epithelium in vivo, and (**D**) stimulated vs. unstimulated IEOs in vitro. Scale bar – 50 μm. IEOs used for western blot analysis and immunohistochemical staining were stimulated with IFNγ + TNF (10 ng/mL IFNγ + 50 ng/mL TNF) for 24 h. UnINF – Uninflamed. INF – Inflamed. Ust – Unstimulated. St – Stimulated.

Our analysis of patient-matched microdissected epithelium and IEOs identified strong concordance in transcriptional responses to inflammation. Therefore, we conducted a patient-specific correlation analysis to investigate the relationship between gene expression levels in vivo and in vitro, using the IFNγ + TNF-stimulated IEOs which best replicated active epithelial UC inflammation. The correlation analysis revealed 250 genes with strong positive correlation (*r* > 0.7) and 142 genes with a strong negative correlation (*r* < -0.7) (Supplementary Fig. 1A). On a global scale, the relationship between in vivo and in vitro expression appeared linear (Supplementary Fig. 1B) and key inflammatory genes were highly correlated (Supplementary Fig. 1C and 1D). To emphasize the patient-specific nature of these responses, we analyzed the paired expression of the positively correlated genes *ERAP2*, important for presentation by MHC class I molecules^26^, and *IRF1*, a transcription factor responsive to inflammatory stimuli^43^. *ERAP2* expression exhibited the donor-specific pattern consistent with the known expression quantitative trait loci (eQTL) effects^26^, with markedly lower expression both in the inflamed epithelia in vivo and the stimulated IEOs in vitro in donors lacking the functional SNP (Fig. 5A). These findings were further validated at protein level by western blot, where one *ERAP2*-expressing donor (D57) showed clear ERAP2 protein induction upon stimulation, whereas another donor (D52) lacking the expression of functional ERAP2 protein (Fig. 5A). These results confirm that the epithelial ERAP2 expression is affected by genotype in IBD and maintained across conditions. Similarly, *IRF1* was significantly upregulated in inflamed versus uninflamed epithelium and in IFNγ + TNF-stimulated versus unstimulated IEOs. Although *IRF1* expression significantly increased during both conditions, the patient-specific order was not preserved (Fig. 5B). Protein level validation (*n* = 4) confirmed robust induction of IRF1 protein in IEOs following IFNγ + TNF-stimulation across donors (Fig. 5B, Supplementary Fig. 2) and increased nuclear IRF1 staining in inflamed epithelial region and IFNγ + TNF-stimulated IEOs, consistent with the transcriptomic data (Fig. 5C, D).

**Fig. 6:**
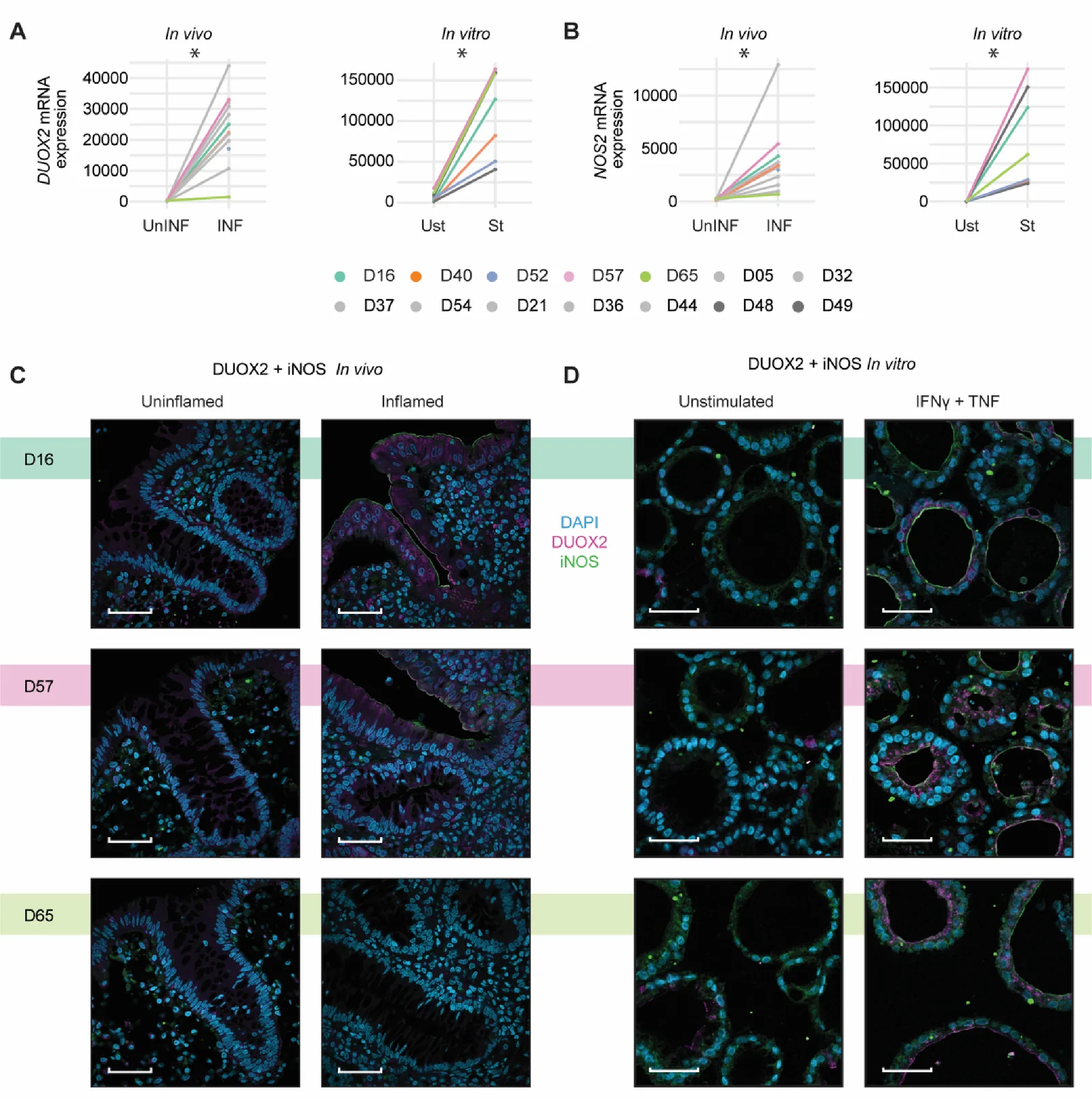
Protein-level responses of DUOX2 and iNOS match transcriptomic expression of *DUOX2* and *NOS2*. Paired comparison of (**A**) *DUOX2* and (**B**) *NOS2* expression levels between uninflamed vs. inflamed epithelium in vivo, and unstimulated vs. IFNγ + TNF-stimulated IEOs in vitro. Each line represents data from the same donor, highlighting within-patient changes. Colored lines represent overlapping in vivo and in vitro donors (*n* = 5), while light grey are in vivo only donors (*n* = 7), and dark grey are in vitro only donors (*n* = 2). Significance values were obtained from differential expression analysis dataset processed by DESeq2 Bioconductor package^31^(* *p* < 0.05). Representative immunofluorescence images from D16, D57, and D65 of DUOX2 (purple) and iNOS (green) showing increase and apical localization in (**C**) inflamed vs. uninflamed colonic epithelium in vivo and (**D**) stimulated vs. unstimulated IEOs in vitro. Scale bar – 50 μm. IEOs used for immunofluorescence staining was stimulated with IFNγ + TNF (10 ng/mL IFNγ + 50 ng/mL TNF) for 24 h. UnINF – Uninflamed. INF – Inflamed. Ust – Unstimulated. St – Stimulated.

We further examined the expression of *DUOX2* (dual oxidase 2) and *NOS2* (inducible nitric oxide synthase) which were consistently upregulated in both in vivo samples and in cytokine-stimulated IEOs (Fig. 4A). NOS2 catalyzes nitric oxide production, while DUOX2 generates hydrogen peroxide, a precursor for antimicrobial reactive oxygen species (ROS)^44^. Dysregulated ROS is implicated in IBD pathogenesis and DUOX2 variants are associated with early onset IBD^45^. Paired analysis of *DUOX2* and *NOS2* expression showed a significant increase in inflamed epithelium compared to uninflamed in vivo and in IFNγ + TNF-stimulated IEOs compared to unstimulated in vitro (Fig. 6A, B). Protein localization of DUOX2 and iNOS protein was confirmed by immunofluorescence staining (*n* = 3), with staining patterns at the apical surface of epithelial cells within the inflamed crypts of colonic tissue and IFNγ + TNF-stimulated IEOs. Protein-levels were similar to the transcriptomic data, showing higher expression of DUOX2 and iNOS in inflamed epithelial region (Fig. 6C) and in IFNγ + TNF stimulated IEOs (Fig. 6D) from donors with elevated mRNA level. Together, these findings demonstrate that IEOs can recapitulate patient-specific epithelial responses associated with UC at both the gene expression and protein-levels, highlighting their potential in precision medicine approach.

## Discussion

Although human IEOs represent a valuable model system for studying development and human cellular mechanisms^24^, there is an ongoing debate about how these organoids should be cultured to replicate the inflammatory phenotype in vivo^25,46,47^. Previous work by Dotti et al.^25^, demonstrated that persistent colonic epithelial changes in UC could originate from dysregulated intestinal stem cell compartment. Previous studies have shown that the transcriptomic difference between pediatric ileal Crohn’s disease and healthy controls are comparable in whole biopsies and matching ileal epithelial organoids shortly after their establishment^48,49^. However, subsequent studies questioned the long-term stability of the inflammatory phenotype, reporting that IEOs from inflamed regions lost their pro-inflammatory signature after four weeks in culture. Consequently, IEOs need re-exposure to inflammatory stimuli to regain their IBD phenotype^46^. Recently, Capeling et al.^50^, developed a physiologically relevant epithelial injury model by characterizing the response of human colon organoids to common stressors, including gamma irradiation, dextran sulphate sodium, mechanical injury, and cytokine stimulation. Among these, a cytokine cocktail of TNF, IFNγ and IL1β were found to most effectively recapitulate IBD-like epithelial gene signatures, whereas conventional stressors induced less disease-relevant responses. A cocktail of 100 ng/mL TNF, 20 ng/mL IL1β and 1 µg/mL flagellin has previously been reported as the optimal mix to reintroduce UC inflammation based on the regulation of *DUOXA2*, *IL1β*, *CXCL8* and *TNF*^46^. In our study the combination of 10 ng/mL IFNγ and 50 ng/mL TNF induced equivalent epithelial processes to the in vivo epithelium. Furthermore, a cocktail of pro- and anti-inflammatory cytokines with low concentrations and a TLR3 agonist also reproduces UC epithelial inflammation in IEOs. Culturing IEOs with lower doses of cytokines could be beneficial if one is interested in avoiding cell death or other unwanted mechanisms induced by high cytokine concentrations. A tenfold reduction in IFNγ concentration led to a distinct change in DEG patterns, which could not be observed with a tenfold reduction in TNF. In rodents, the TNF concentrations fluctuation between homeostasis and inflammation is more than 10-fold change^10^, which could explain the lack of difference between our low and high TNF concentration.

Our study demonstrated that IEOs, when stimulated with IFNγ + TNF, recapitulated key transcriptional and protein-level features of inflamed colonic epithelium in UC. This is particularly significant given the molecular heterogeneity of IBD^51^, which demands model systems that can recapitulate the patient-specific inflammatory mechanisms and cellular responses^52^. Central to this challenge is establishing that patient-specific cellular responses and molecular signatures observed in vitro consistently mirror the in vivo disease state. Our paired and patient-matched transcriptomes represent a unique opportunity to study the individual preservation of gene regulation. The strong correlation shown here between the in vivo and in vitro gene expression profile supports the notion that IEOs conserved several inflammatory transcriptional processes during inflammation. A key example of genotype-dependent regulation is *ERAP2*, whose expression was tightly linked to the rs2910686 SNP. The present findings align with our recent work demonstrating that *ERAP2* expression in IBD organoids is upregulated upon IFNγ stimulation only in donors with the functional rs2910686 allele, while rs2910686 TT homozygotes showed no induction. This genotype-dependent expression was preserved across in vivo and in vitro condition, suggesting the utility of IEOs in studying eQTL effects in epithelial inflammation^26^.

IRF1, a central transcription factor involved in both JAK-STAT and NFκB signaling^43^, plays a pivotal role in orchestrating inflammatory response in IBD. In our study, IRF1 was upregulated in both inflamed epithelium and IFNγ + TNF-stimulated IEOs, supporting its role as a conserved inflammatory regulator. The high correlation of intracellular signaling genes such as *IRF1*,* IRAK2*,* CARD6* further indicates that UC-specific processes are faithfully recapitulated in vitro. We also observed consistent upregulation of DUOX2 and iNOS, enzymes involved in ROS production^44^. Increased iNOS expression in epithelial cells could suggest a potential role in activating the proposed regulation of PANoptosis through JAK-STAT-IRF1-iNOS-NO axis^53^. This activation could further amplify inflammatory response. Our data confirm that IEOs can model UC-associated inflammatory signaling and oxidative stress responses with high fidelity, showing transcriptomic and protein-level concordance between inflamed tissue and stimulated organoids.

Inter-individual variation observed between patient-derived samples likely reflects the biological heterogeneity inherent to human intestinal tissue, including differences in the disease state and genetic background between donors. This variability represents strength of the model, further supporting the physiological relevance of the IEO model compared to homogeneous cell lines. Nevertheless, IEOs represent a simplified model to study epithelial functions while lacking the complex cellular and molecular interactions present in the in vivo environment such as platelet-endothelium-leucocyte interactions, complement system and chemotaxis. This limitation likely explains the absence of *IL16* and *CCL11* regulation in vitro, despite their upregulation in active UC and CD^54^. Although TNF has been shown to induce *CCL11* in bronchial epithelium^55^, we did not observe this response in IEOs, suggesting tissue-specific regulatory mechanisms. Alternatively, the epithelial compartment regulation of *CCL11* and *IL16* could be from intra epithelial lymphocytes^56^, which are not present in IEOs.

The strength of this study is the unique nature of patient-matched in vivo and in vitro transcriptomes and protein-level validation of key inflammatory genes offering the ability to evaluate IEOs as a model system for UC and the donor-adjusted transferability of gene expression. A limitation of this study is the fixed stimulation duration (8 h for IFN/TNF and 24 h for the cytokine cocktail), which captures early and delayed primary response genes^57^ but does not reflect the chronic, dynamic stimulation present in vivo. A temporal gene expression analysis could have provided additional insights into the transcriptional program associated with sustained inflammation. However, longer stimulation would likely introduce secondary autocrine and paracrine effects, complicating interpretation in the IEO model. Another limitation of the correlation analysis between in vivo and in vitro transcriptomes is the small sample size, which impacts statistical power and estimate precision with increased risk of false positives. The sample size also affects the differential gene expression and enrichment analysis with increased sensitivity to outliers and risk of type two errors that may mask moderate biological associations. While our analysis provides initial insights into gene expression patterns during inflammation, validation through larger cohorts would strengthen these findings. Transitioning to precision medicine entails interpreting results from a single or small group of patients. Therefore, the sample size, although small, is reflective of the challenge posed by precision medicine. However, to build confidence in the correlation of in vitro models to in vivo functions one initially needs larger sample sizes.

## Conclusion

Cytokine-stimulated IEOs acquired from uninflamed colonic areas successfully replicate active UC epithelial inflammation. Especially the combination of IFNγ + TNF or a cocktail of Poly(I:C), TNF, IFNγ, IL1β, IL17 and IL22 induced UC epithelial inflammatory responses. Importantly, we provide a reference dataset of transcriptomic changes during epithelial inflammation both in vivo and in an in vitro IEO model from the same UC patient group. Our patient-matched, donor-specific correlation analysis provides a framework for evaluating epithelial gene expression across in vivo and in vitro systems, strengthening the use of IEOs as human preclinical model.

## Supplementary Information

Below is the link to the electronic supplementary material.


Supplementary Figure 1



Supplementary Figure 2



Supplementary Figure legend


## Data Availability

The RNA sequencing datasets generated and analyzed during the current study are available in the NCBI´s Gene Expression Omnibus (GEO) repository and are accessible through GEO Series accession numbers GSE288617, GSE288517, GSE289072. All supplementary materials, including data files and detailed metadata descriptions and original code has been deposited at Zenodo and are publicly available at https://zenodo.org/records/19912112. Any additional information required to reanalyze the data reported in this paper is available from the lead contact upon request.

## Abbreviations

BSA: Bovine serum albumin
CXCL: Chemokine ligand
DAPI: 4′,6-diamidino-2-phenylindole
DEG: Differentially expressed genes
DPBS: Dulbecco’s phosphate-buffered saline
dTTP: Deoxythymidine triphosphate
DUOX2: Dual oxidase 2
dUTP: Deoxyuridine triphosphate
eQTL: Expression quantitative trait locus
ERAP2: Endoplasmic reticulum aminopeptidase 2
FFPE: Formalin-fixed paraffin-embedded
GAPDH: Glyceraldehyde-3-phosphate dehydrogenase
IBD: Inflammatory bowel disease
IEO: Intestinal epithelial organoid
IFN: Interferon
IL: Interleukin
IRF1: Interferon regulatory factor 1
LMD: Laser microdissection
NFκB: Nuclear factor kappa-light-chain-enhancer of activated B cells
NOS2: Nitric oxide synthase
Poly(I: C) Polyinosinic: polycytidylic acid
TBST: Tris-buffered saline with Tween
TLR: Toll-like receptor
TNF: Tumor necrosis factor
UC: Ulcerative colitis
