# Supplementary material for: Patient-matched transcriptomics of in vivo and in vitro colonic epithelium substantiate organoids as a translational model for ulcerative colitis
Source: Sci Rep. 2026 Jun 14;16:27171. doi: 10.1038/s41598-026-54857-7 (PMC13527059; doi:10.1038/s41598-026-54857-7)
Supplement: Supplementary file 1 [file 41598_2026_54857_MOESM3_ESM.docx]

# Supplementary Figure legends

**Supplementary Figure 1. UC-relevant gene expression levels *in vivo* correlated with *in vitro* at patient-specific resolution** (**A**) Histogram displaying the correlation coefficient of each gene (n = 1840) shared between the gene list of differentially expressed genes (DEGs) *in vivo* (inflamed *vs* uninflamed) and IFNγ + TNF-stimulation of intestinal epithelial organoids (IEOs) (stimulated *vs* unstimulated). Binwidth = 0.1. The orange bins represent genes with a correlation coefficient ≥ 0.7 / ≤ -0.7 which were included in subsequent analyses. (**B**) Hexagonal binning plot illustrating the correlation between *in vivo* and *in vitro* gene expression patterns for genes identified in panel **A**. The x-axis represents variance-stabilizing transformed (VST) gene expression values from inflamed epithelium (*in vivo*), while the y-axis shows VST gene expression values from IFNγ + TNF-stimulated IEOs (*in vitro*). Each hexagon represents a distinct spatial bin (bin size = 70), with color intensity indicating the density of genes within that region. Brighter colors denote higher numbers of donor-specific genes falling within each hexagonal area. (**C, D**) Scatter plots of genes with a correlation coefficient ≥ 0.7 (**C**) or ≤ -0.7 (**D**). The x-axis represents VST gene expression values from inflamed epithelium (*in vivo*), while the y-axis shows VST gene expression values from IFNγ + TNF-stimulated IEOs (*in vitro*). A subset of genes is colored and named, and a linear regression curve is fitted to each gene based on the donor-specific gene values.

**Supplementary Figure 2.** Full blot images from all the replicates of intestinal epithelial organoids (IEOs) stimulated with IFNγ + TNF, showing IRF1 and ERAP2 expression corresponding to data presented in Figure 5.
